# Plant diversity and community composition in managed humid coastal dune slacks in NW England

**DOI:** 10.1371/journal.pone.0256215

**Published:** 2021-08-19

**Authors:** Margaret A. Connor, Stephanie Tarvin, Megan Quail, Sven Peter Batke

**Affiliations:** Biology Department, Edge Hill University, Ormskirk, United Kingdom; Chinese Academy of Forestry, CHINA

## Abstract

Humid coastal dune slacks are an endangered habitat in Northwestern Europe. In the UK, dune slacks are currently classified as being in ‘unfavourable’ condition, with projected decrease in England of up to 30% by 2036. Studies in mainland Europe suggest that regional factors (e.g. slack area, age, and isolation) are more important than local factors (e.g. soil, pH, soil nutrient and water status) in driving successional vegetation processes in coastal slacks. However, this has never been tested for the UK, where approximately 14% of European slacks occur. We used previously established survey protocols to test whether regional factors are more important than local factors in UK coastal slacks, along the Sefton Coast in NW England. We found that slack area and slack age were more important than local factors in driving plant community composition and species richness. We also showed that higher levels of management, such as active grazing and invasive shrub and tree removal, are effective in increasing soil moisture levels in slacks. Our results suggest that similar successional processes are likely to be important in slacks in the NW of England, compared to mainland Europe.

## Introduction

Humid dune slacks are low-lying wetland coastal habitats that are subjected to large seasonal shifts in the water table. Approximately 14% of all European slacks can be found in the UK; of which 30% within England are predicted to disappear by 2036 [1]. Further projections suggest that adverse climate conditions, such as changes in local precipitation levels, will lead to the decline and replacement of slack communities by 2050 with grassland communities [2]. Many slacks in the UK are currently classified as ‘unfavourable’, due to land development, drainage and under-grazing [3]. As a result, many rare and endemic species that are often associated with coastal slacks are becoming more threatened [4].

Key local factors that are believed to determine changes in plant community composition and succession in dune slacks include biomass accumulation over time [5–7], seed bank age [8, 9], soil moisture, pH and hydrological changes [6, 10, 11] and management intensity such as cutting and grazing [12]. For example, it has been shown that soil accumulation and scrub encroachment can result in an increase in litter, dead organic matter and shading [5, 13], thus negatively altering soil mineral levels [7] and resulting in a decrease in species diversity over time. Many of these local factors, however, are believed to be less important at a regional scale, where dune slack age, slack area and slack isolation are believed to be of greater importance in shaping plant community assemblages [14]. It has been shown that older and larger slacks have different vegetation communities, compared to smaller and younger slacks. This has been demonstrated both for slacks in mainland Europe and Asia [14, 15]. Slack isolation (i.e. the distance to the nearest neighbouring slack) is believed to affect succession rates, with more isolated slacks being colonised more slowly than less isolated slacks [14].

In the UK, there has only been a small number of studies investigating the effect of local and regional factors on plant community diversity and successional processes in humid coastal slacks [12, 16]. In contrast, dune slacks elsewhere in Europe have received comparatively more attention [5–11, 14]. For example, Bossuyt, Honnay [14] investigated 83 coastal slacks in France and Belgium, studying the effect of both regional (age, area, isolation) and local factors (dispersal strategy or MIV for light, soil pH, moisture and nitrogen) on plant successional processes. They found that although local factors are somewhat important in determining the community composition of plants in slacks, slack age, area, and isolation can strongly limit the effect of local factors due to changes in the successional process. However, it remains to be seen whether these results can be applicable to other slack systems elsewhere in Europe.

Using the same methodology employed by Bossuyt, Honnay [14], we investigated and compared for the first time, whether similar local and regional factors are important in driving plant community and diversity differences in coastal dune slacks on the Sefton Coast in NW England—a Special Area of Conservation (SAC) and a Site of Special Scientific Interest (SSSI). Our specific aims were to compare the diversity and community composition of dune slacks across a chronosequence along the coast of NW England and to identify whether the community composition and richness of slacks differed with slack isolation, area, slack management and environmental slack characteristics.

## Materials and methods

The study was scrutinized and approved under the Edge Hill University ethics board and code of conduct.

### Study sites and design

**Fig 1:**
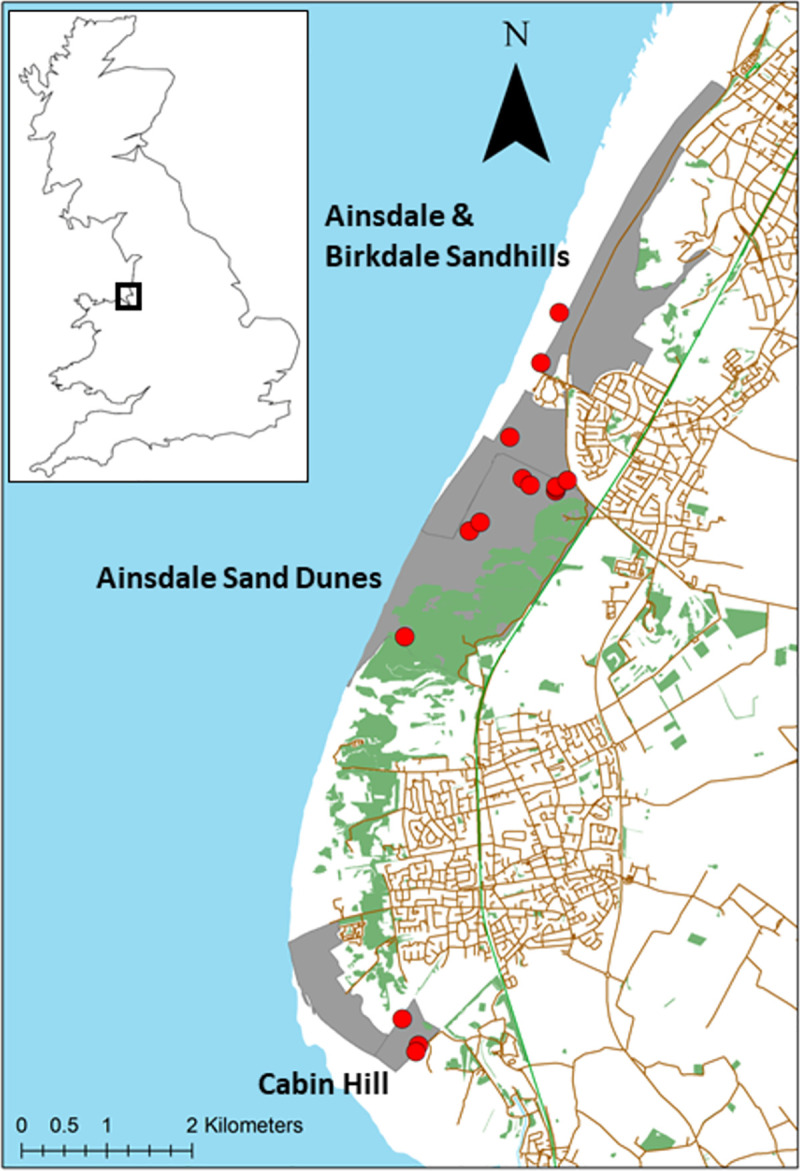
Sample locations in NW England. Blue points mark individual slacks (n = 15). Base map was used and modified from Natural England Open Data.

A total of 121 1x1m quadrats from 15 humid slacks were surveyed between May and July in 2019. The number of quadrats per slack varied with the size of slacks and followed the protocol by Bossuyt, Honnay [14]. The minimum number of quadrats per slack was five and the maximum was 20. The slacks were surveyed within three nature reserves, namely Ainsdale Sand Dunes National Nature Reserve (508 ha), Cabin Hill National Nature Reserve (30 ha) and Ainsdale and Birkdale Sandhills Local Nature Reserve (296 ha) (Fig 1). Ainsdale Sand Dunes and Cabin Hill are managed by Natural England and the Ainsdale and Birkdale Sandhills Local Nature Reserve is managed by Sefton Council. All three sites are designated as SSSIs. Current management of the dune slack vegetation aims to keep them in ‘favourable condition’, mostly by removal of invasive, woody scrub and trees, grazing by livestock and wild rabbits, mowing and occasional reprofiling (S1 Table). We classed all slacks into four management levels (no, low, medium, and high). Low was defined by rabbit grazing and occasional scrub removal, medium was defined by annual winter-grazing, rabbit grazing and occasional scrub removal and high was defined by annual winter-grazing, regular mowing and/or scrub removal and rabbit grazing (S1 Table).

### Data collection

Within each slack, quadrats were randomly placed, and all vascular plant species were identified using several different identification guides [17–20]. Abundance for each species and percentage of bare ground was estimated to the nearest 5% percentage for each quadrat by the same surveyor. Using the LEDA Traitbase [21], we separated species into different seed dispersal groups following Bossuyt, Honnay [14]. The mean relative cover of anemochorous, autochorous, endozoochorous, epizoochorous, hydrochorous, myrmecochorous and hemerochorous species was calculated for each quadrat by dividing the sum of the cover of each species, for each dispersal group, by the total vegetation cover of the quadrat.

Soil moisture was measured at three location within each quadrat using a *Procheck* hand-held moisture meter (n = 363). Soil pH for each slack was measured with a hand-held pH meter. In addition, mean Ellenberg indicator values (MIVs) for soil nitrogen and light were calculated for each species for each quadrat and slack using the PLANTATT database [22]. Slack age was defined as the number of years since the slack was last reprofiled or scraped. Slack isolation was defined by measuring the mean distance of each slack centre, using ArcGIS, to the three nearest slack neighbours following Bossuyt, Honnay [14].

### Data analysis

The community data was visualised using Multidimensional Scaling (MDS) with the R ‘vegan’ package [23]. To identify the most important response variable that affected species richness, abundance and community composition (i.e. MDS scores), the explanatory variables were correlated in a random/mixed-effects meta-regression model with slack area, slack isolation, slack age, mean and maximum soil moisture (S.M.), percentage bare ground and soil pH as response variables. We used the ‘glmulti’ package in R for this analysis [23]. We fitted the meta-regression model separately for MDS axis 1 and 2. The best-fit model was selected using Akaike’s Information Criterion (AIC). A generalised linear model was used to determine the correlation coefficient for response and explanatory variable combinations. A Kruskal-Wallis test was used to identify how response variables at a slack level differed between past management levels. In addition, Partial Pearson analysis was used to test whether the frequency of species in different dispersal groups correlated with slack age, slack isolation, or slack area. All analysis was performed in R [23].

In order to investigate the mean interaction between slack age, species richness and slack area, and slack age, abundance and slack isolation, we combined our data (n = 15) with that from Bossuyt, Honnay [14]. Data was directly extracted from their published article, by digitising relevant figures. Mean slack richness and abundance data was available for 83 slacks for sites in France and Belgium. A visual comparison was made, as the available data did not allow satisfactorily statistical analysis.

## Results and discussion

**Fig 2:**
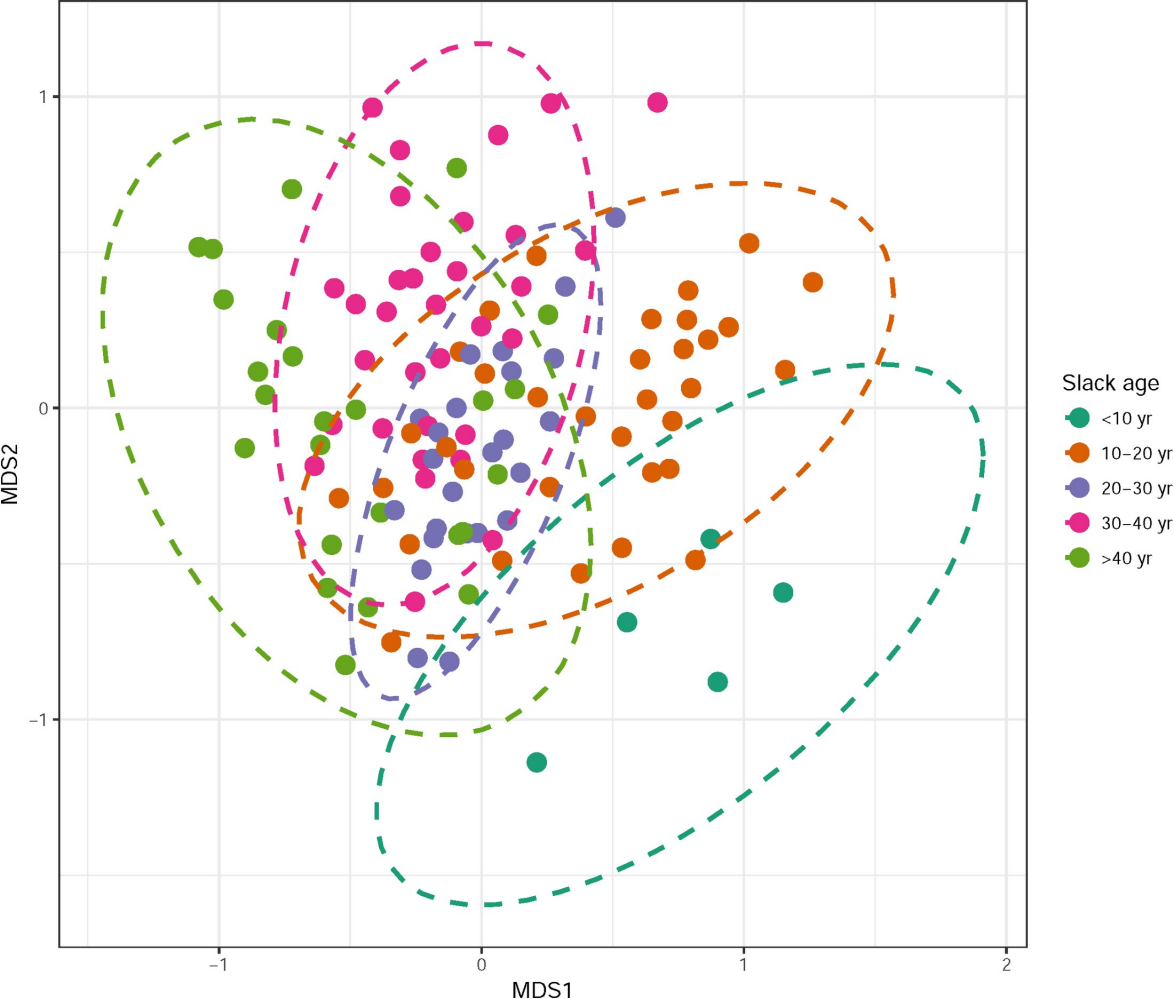
Multidimensional scaling (MDS) ordination. Scores are plotted for each quadrat (n = 121), colour-coded and grouped by slack age.

A total of 110 different plant species were recorded in 121 quadrats across 15 slacks. Fourteen of these species were of conservation importance (13%—S2 Table). The frequency of these 14 species did not differ between slack age, isolation, area, or level of management (not shown). Species richness was positively associated with an increase in slack area and negatively with maximum soil moisture and percentage of bare ground (Table 1). Abundance was negatively associated with slack isolation and percentage of bare ground (Table 1). The MDS analysis suggested a two-dimensional interpretation of the community data with good ties (stress = 0.19). Overall, MDS axis 1 explained most of the clustering in the community data (Fig 2) and was negatively associated with slack age and negatively associated with maximum soil moisture (Table 1). A Kruskal-Wallis test showed that maximum soil moisture was higher in older slacks compared to younger slacks (Chi-squared = 17.445, df = 4, p-value = 0.001584). MDS axis 2 was weak, positively associated with slack area and negatively with soil pH (Table 1). There were no differences in the frequency of species that had different dispersal strategies when compared to slack age, slack isolation, or slack area (Partial Pearson correlation = >0.05).

**Table 1:** Random/mixed-effects meta-regression model results for the best fit models.

	Richness	Abundance	MDS 1	MDS 2
**Best-fit model**	~ 1 + Bare ground (%) + Area + Max. S.M.	~ 1 + Bare ground (%) + Isolation	~ 1 + Bare ground (%) + Area + Age + Max. S.M. + pH	~ 1 + Bare ground (%) + Area + Age + Mean S.M. + pH
**AIC (R** ^**2**^ **)**	597.6 (0.30)	881.7 (0.45)	17.6 (0.75)	75.2 (0.42)
Slack area	4.59***(0.21)	Ns	6.18***(0.10)	4.59***(0.14)
Slack isolation	Ns	-2.088*(0.02)	ns	Ns
Slack age	Ns	ns	-8.474***(0.50)	4.469***(0.04)
Mean S.M.	Ns	ns	ns	-6.743***(0.11)
Max. S.M.	-3.323**(0.10)	ns	-7.64***(0.31)	Ns
Bare ground (%)	-2.199*(0.10)	-9.557***(0.43)	3.25**(0.10)	-2.199*(0.02)
Soil pH	Ns	ns	6.119***(0.10)	-2.732**(0.14)

MDS axis 1 and 2, species richness and abundance were modelled as a response variable for different explanatory variables. AIC was used to select the best fit model for each response variable. A generalized linear model was used separately for each response and explanatory variable to determine the correlation coefficient (R^2^), which is shown in brackets. Significant codes:

<0.001***

0.001**

0.01*

0.05^(.)^, >0.05 (ns not significant).

Richness, abundance, MDS 1, MDS 2, percentage of bare ground, MIV light, MIV soil nitrogen and soil pH did not differ statistically between different levels of slack management (Kruskal-Wallis = >0.05). However, mean (Chi^2^ = 7.892, df = 3, p = 0.05) and maximum soil moisture content (Chi^2^ = 9.067, df = 3, p = 0.03) did differ between management levels. Slacks that received low or no management, were statistically different from medium and highly managed slacks [mean soil moisture (t-value = -3.28, p<0.01) and maximum soil moisture (t-value = -2.97, p<0.01)].

**Fig 3:**
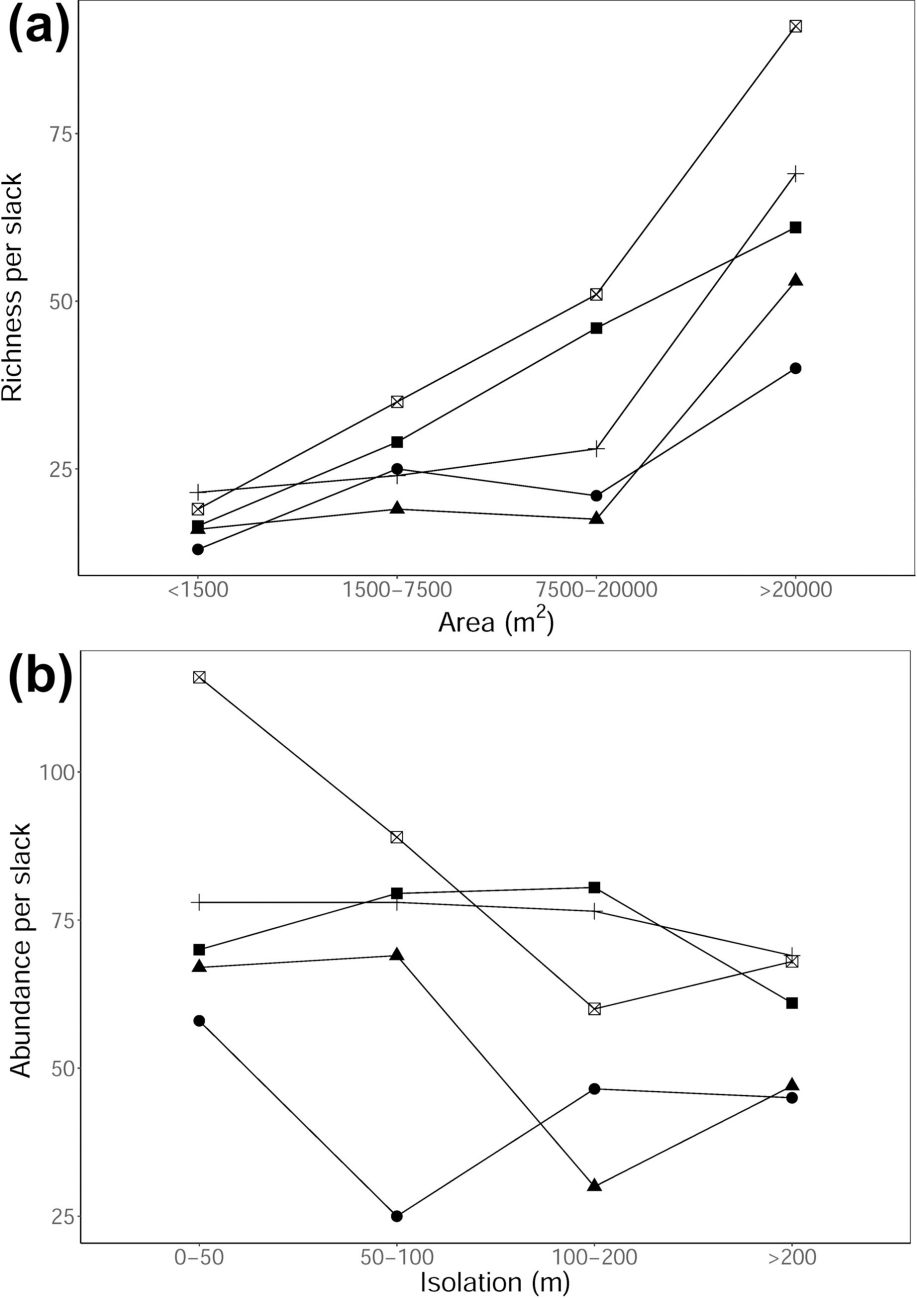
Interaction between age and (a) species richness and slack area, and age and (b) abundance and slack isolation for the plot mean combined data from Bossuyt, Honnay [14] and this study. The different symbols represent different slack ages. Circles = <5 yrs., triangles = 5–15 yrs., solid squares = 15–25 yrs., pluses = 25–50 yrs., and squares with a diagonal cross = >50 yrs.

When we combined our data with the mean slack species richness data of Bossuyt, Honnay [14], we found consistent results, that larger slacks had a higher mean species richness (Fig 3A). In addition, the combined data showed that mean species richness was significantly higher in older slacks. We further found that mean abundance decreased with isolation when combing these two data sets and that like richness, older slacks had a higher mean abundance (Fig 3B).

Our results showed that there are significant changes in humid dune slack plant communities during forty years of succession on the Sefton Coast of NW England. Regional factors such as slack age and slack area were more important factors than local ones (e.g. soil pH, soil moisture and MIV for light and nutrient soil status). For example, we found a positive correlation between species richness and slack area, which has also been reported in other parts of mainland Europe [14] and Asia [15]. The lower number of species often observed in smaller slacks is likely the result of increased edge-effects [24, 25], making them more susceptible to changes in external environmental conditions [26]. For instance, when combining our data with Bossuyt, Honnay [14], we found that the difference in plant species richness between age categories was substantially higher in larger slacks compared to smaller ones. This evidence is indicative of a successional pathway, whereby older and larger slacks can support a higher number of species due to more extensive time periods for colonisation, whilst also providing more favourable environmental conditions for establishment. However, opposing to Berendse, Lammerts [5], and when joining our data with Bossuyt, Honnay [14], we found that the smallest slacks mostly did not have a higher species richness compared to some of the larger slacks, indicating that small slacks are less likely to be important seed sources for a high number of slack species.

Comparable to the results of Bossuyt, Honnay [14], we found that species abundance was lower in more isolated slacks. It is likely that the rate of succession and biomass accumulation in more isolated slacks is lower, due to a slower colonisation rate and lower numbers of autochorous species [14]. In addition, and only when combining our data with Bossuyt, Honnay [14], we found that older, more isolated slacks had a similar species abundance compared to younger, less isolated slacks. However, the data was not very consistent. It has been suggested that establishment and succession on more isolated dune slacks appears to be limited by dispersal mode and the availability of dispersal agents (e.g. grazing animals, flooding) [6, 9]. For example, it has been shown that in young isolated slacks the substrate is more favourable for the establishment of small pioneer species, whereas substrate conditions continue to become less favourable following the arrival of dispersed seeds from early colonisers [8]. During early successsional processes recruitment from the seed bank and establishment from wind dispersed species is most likely more important [27]. Hence why community composition in our slacks did not differ between slacks that were more or less isolated (Table 1). However, slacks that are larger and older represent an increased successional stage, and thus it is likely that dispersal from outside the slack becomes more important. For example, we found, and comparable with Bossuyt, Honnay [14], that the community difference between slacks increased with increasing slack age. It is believed that particularly during early successsional processes the soil conditions (moisture and nutirents) can limit the number of pioneer species able to establish. With increasing age and area, species composition is thererfore less depenedent on deterministic habitat conditions, but is more affected by distance to dispersal sources [14]. Although, in our study the frequency of species with different dispersal modes did not differ between slacks of different ages and isolation. It is likely that the closer proximity of the slacks in our study compared to Bossuyt, Honnay [14] study, masked the effect of the importance of different dispersal modes. This highlights the importance of the landscape configuration of slacks during successional processes, as changes in community composition in older slacks are often smaller, particularly when slacks are more isolated or small [14].

Our ordination analysis demonstrated that slack age and maximum soil moisture were the most important variables along MDS axis 1 (R^2^ = 0.75). Similar results have been reported in Northern Europe, with soil moisture being identified as an important contributing factor to plant composition gradients [10]. Specifically, our study showed that maximum soil moisture was significantly higher in older slacks and in those that received high levels of management (annual winter-grazing, regular mowing and/or scrub removal and rabbit grazing). In contrast, soil moisture was lower in younger slacks and those that received no or low levels of management (rabbit grazing and occasional scrub removal). This is not surprising given that current local management initiatives include the removal of invasive shrubs and trees [12], with priorities to maintain or increase the water-table in the slack [11, 28]. These findings are comparable with those of Bossuyt, Honnay [14] in Belgium and France, who showed a significant increase in MIV soil moisture with slack age. However, slack management was not directly assessed and only slacks up to an age of 20 years were under management in their study [14], making a comparison difficult.

## Conclusions

In conclusion, our study along the Sefton Coast of NW England confirmed that similar local and regional successional factors are acting on dune slack plant communities, when compared to other humid dune slacks in mainland Europe. Slack area, age and to some degree slack isolation and management have been shown to drive successional pathways and appear to be more important than local deterministic habitat conditions in the NW of England.

## Supporting information

S1 Table Current and past management of the 15 studied dune slacks. Historical data was obtained through *The Dune Wetlands Project* [28] and from searches of the Ainsdale Sand Dunes National Nature Reserve archives. Data of current management was obtained via personal communication with the site managers. No records were available for slacks over 50 years old. ND = no data.
(DOCX)

S2 Table List of species and their conservation status. Data collected from across 15 slacks in Ainsdale Sand Dunes National Nature Reserve, Cabin Hill National Nature Reserve and Ainsdale and Birkdale Sandhills Local Nature Reserve. CI = Species of Conservation Importance in North West England and NS = Nationally Scarce.
(DOCX)
