# Supplementary material for: Plant diversity and community composition in managed humid coastal dune slacks in NW England
Source: PLoS One. 2021 Aug 19;16(8):e0256215. doi: 10.1371/journal.pone.0256215 (PMC8375971; doi:10.1371/journal.pone.0256215)
Supplement: S1 Table — Historical data was obtained through The Dune Wetlands Project [28] and from searches of the Ainsdale Sand Dunes National Nature Reserve archives. Data of current management was obtained via personal communication with the site managers. No records were available for slacks over 50 years old. ND = no data. (DOCX) [file pone.0256215.s001.docx]

**S1 Table. Current and past management of the 15 studied dune slacks.** Historical data was obtained through *The Dune Wetlands Project* [1] and from searches of the Ainsdale Sand Dunes National Nature Reserve archives. Data of current management was obtained via personal communication with the site managers. No records were available for slacks over 50 years old. ND = no data.

|  | **1976-2007 data** | | | | | **Current data (2019)** |
| --- | --- | --- | --- | --- | --- | --- |
| **Site & slack no.** | **Description (2007)** | **Mowing** | **Grazing** | **Scrub clearance** | **Reprofiling** | **Management** |
| **Cabin Hill National Nature Reserve** | | | | | | |
| 1a | Borrow-pits + scrapes (1976) | 1993 (a) 1994 1995 1998 | Before 1991, horses, cattle, sheep from 1992 | 1989, 1990 | 1984 1988 1990 1991 2006 | Drier section mown 2018.  Annually winter--grazed by sheep and cattle. |
| 25 | Mature dry slack (now humid) | ND | ND | ND | ND | Removal of scrub and trees 2018. Annually winter-grazed by sheep and cattle. |
| 26 | Mature dry slack (now humid) | ND | ND | ND | ND | Removal of scrub and trees 2018. Half the slack is annually winter-grazed by sheep and cattle. |
| **Ainsdale Sand dunes National Nature Reserve** | | | | | | |
| 8 | Mature wet-slack (scrape 1977) | NA | Rabbits | 2002 2003 | 2004 | Removal of sea buckthorn 2012. Grazed by rabbits |
| 49 | Scrape 1977 | 2003 2005 | Sheep and cattle | ND | 1992 part infilled | Mown in the last 10 years. Annually winter-grazed by sheep and cattle. |
| 53 | Scrape 1976 | NA | Sheep from 1991 | ND | 1991 reprofiled | Scrub clearance 2011  Annually winter-grazed by sheep and cattle. |
| 103 | Mature wet-slack (scrape 1976) | ND | Sheep from 1991 | ND | None | Annually winter-grazed by sheep and cattle. |
| 104 | Mature wet-slack (scrape 1976) | NA | Sheep from 1991 | NA | 1993 | Annually winter-grazed by sheep and cattle. Mown occasionally. |
| 143a | Dry-slack (now humid) | NA | Rabbits | NA | 1990 | Occasional removal of sea buckthorn and pine. Grazed by rabbits. |
| 143b | Dry-slack (now humid) | ND | Rabbits | ND | ND | Occasional removal of sea buckthorn and pine. Grazed by rabbits. |
| 144 | Dry-slack (now humid) | ND | Rabbits | ND | ND | Occasional removal of sea buckthorn and pine. Grazed by rabbits. |
| **Ainsdale and Birkdale Sandhills Local Nature Reserve** | | | | | | |
| Natterjack scrape (NScr) | NA | NA | NA | NA | NA | Located in frontal dunes. Excavated 2012 |
| Birkdale New Green Beach (NGB) | NA | NA | NA | NA | NA | Formerly a beach car park. Colonisation by plants began 2005. |
| 169 | Incipient wet-slack | 1997 | ND | 1988/89 2000 | NA | Several small scrapes excavated in last 2 years but not sampled. Grazed by rabbits, trampled by humans. |
| 181 | Sand extraction site (scrape 2000) | ND | ND | NA | 2000 | Annually winter-grazed by cattle. |
