# Supplementary material for: Plant diversity and community composition in managed humid coastal dune slacks in NW England
Source: PLoS One. 2021 Aug 19;16(8):e0256215. doi: 10.1371/journal.pone.0256215 (PMC8375971; doi:10.1371/journal.pone.0256215)
Supplement: S2 Table — Data collected from across 15 slacks in Ainsdale Sand Dunes National Nature Reserve, Cabin Hill National Nature Reserve and Ainsdale and Birkdale Sandhills Local Nature Reserve. CI = Species of Conservation Importance in North West England and NS = Nationally Scarce. (DOCX) [file pone.0256215.s002.docx]

**S2 Table. List of species and their conservation status**. Data collected from across 15 slacks in Ainsdale Sand Dunes National Nature Reserve, Cabin Hill National Nature Reserve and Ainsdale and Birkdale Sandhills Local Nature Reserve. CI = Species of Conservation Importance in North West England and NS = Nationally Scarce.

| **Taxonomic name** | **Conservation status** |
| --- | --- |
| Ophioglossaceae |  |
| *Ophioglossum vulgatum* L. | CI |
| Betulaceae |  |
| *Alnus glutinosa* (L.) Gaertn. |  |
| Polygonaceae |  |
| *Persicaria amphibia* (L.) Delarbre |  |
| Polygalaceae |  |
| *Polygala vulgaris* L. |  |
| Fabaceae |  |
| *Anthyllis vulneraria* L. |  |
| *Lathyrus pratensis* L. |  |
| *Lotus corniculatus* L. |  |
| *Lotus pedunculatus* Cav. |  |
| *Medicago lupulina* L. |  |
| *Ononis repens* L. |  |
| *Trifolium* sp.1 |  |
| *Trifolium pratense* L. |  |
| *Trifolium repens* L. |  |
| *Vicia* sp.1 |  |
| *Viciacracca* L. |  |
| Primulaceae |  |
| *Anagallis tenella* (L.) L. | CI |
| *Lysimachia maritima* (L.) Galasso, Banfi & Soldano |  |
| *Samolus valerandi* L. | CI |
| Cyperaceae |  |
| *Bolboschoenus maritimus* (L.) Palla |  |
| *Blysmus compressus* (L.) Panz. ex Link | CI |
| *Carex arenaria* L. |  |
| *Carex flacca* Schreb. |  |
| *Carex hirta* L. |  |
| *Carex nigra* (L.) Reichard |  |
| *Carex oederi* Retz. |  |
| *Eleocharis palustris* (L.) Roem. & Schult. |  |
| *Eleocharis quinqueflora* (Hartmann) O. Schwarz | CI |
| *Isolepis setacea* (L.) R. Br. |  |
| Ranunculaceae |  |
| *Caltha palustris* L. |  |
| *Ranunculus acris* L. |  |
| *Ranunculus aquatilis* L. |  |
| *Ranunculus bulbosus* L. |  |
| *Ranunculus flammula* L. |  |
| *Ranunculus repens* L. |  |
| Asteraceae |  |
| *Bellis perennis* L. |  |
| *Cirsium arvense* (L.) Scop. |  |
| *Hieracium* sp.1 |  |
| *Hypochaeris radicata* L. |  |
| *Leontodon saxatilis* Lam. |  |
| *Leontodon* sp.1 |  |
| *Pilosella officinarum* F.W. Schultz & Sch. Bip. |  |
| *Pulicaria dysenterica* (L.) Bernh. |  |
| *Senecio jacobaea* L. |  |
| *Sonchus asper* (L.) Hill |  |
| *Sonchus oleraceus* L. |  |
| *Taraxa cumofficinale* F.H. Wigg. |  |
| Poaceae |  |
| *Agrostis capillaris* L. |  |
| *Agrostis stolonifera* L. |  |
| *Ammophila arenaria* (L.) Link |  |
| *Anthoxanthum odoratum* L. |  |
| *Dactylis glomerata* L. |  |
| *Holcus lanatus* L. |  |
| *Phleum arenarium* L. |  |
| *Phragmites australis* (Cav.) Trin. ex Steud. |  |
| *Schedonorus arundinaceus* (Schreb.) Dumort. |  |
| Gentianaceae |  |
| *Blackstonia perfoliata* (L.) Huds. |  |
| *Centaurium erythraea* Rafn |  |
| *Centaurium littorale* (Turner) Gilmour | NS/CI |
| Caryophyllaceae |  |
| *Cerastium fontanum* Baumg. |  |
| *Sagina nodosa* (L.) Fenzl |  |
| *Stellaria graminea* L. |  |
| Orchidaceae |  |
| *Anacamptis pyramidalis* Rich. | CI |
| *Dactylorhiz afuchsii* (Druce) Soó |  |
| *Dactylorhiz aincarnata* (L.) Soό | NS/CI |
| *Dactylorhiz apraetermissa* (Druce) Soó |  |
| *Dactylorhiz apurpurella* (T. Stephenson & T.A. Stephenson) Soó | CI |
| *Epipactis palustris* (L.) Crantz | CI |
| Juncaceae |  |
| *Juncus articulates* L. |  |
| *Juncus bufonius* L. |  |
| *Juncus compressus* Jacq. |  |
| *Juncus conglomeratus* L. |  |
| *Juncus gerardii* Loisel. |  |
| *Juncus inflexus* L. |  |
| Rosaceae |  |
| *Potentilla anserina* L. |  |
| *Potentilla reptans* L. |  |
| *Rubus caesius* L. |  |
| Salicaceae |  |
| *Salix caprea* L. |  |
| *Salix cinerea* L. |  |
| *Salix repens* L. |  |
| Brassicaceae |  |
| *Cardamine pratensis* L. |  |
| Onagraceae |  |
| *Chamerion angustifolium* (L.) Holub |  |
| *Oenothera* sp.1 |  |
| Orobanchaceae |  |
| *Euphrasia* sp.1 |  |
| *Rhinanthus minor* L. |  |
| Linaceae |  |
| *Linum catharticum* L. |  |
| Equisetaceae |  |
| *Equisetum arvense* L. |  |
| *Equisetum fluviatile* L. |  |
| *Equisetum palustre* L. |  |
| *Equisetum variegatum* Schleich. ex F. Weber & D. Mohr | NS/CI |
| Boraginaceae |  |
| *Myosotis* sp.1 |  |
| *Myosotis laxa* Lehm. |  |
| Asparagaceae |  |
| *Asparagus officinalis* L. |  |
| Plantaginaceae |  |
| *Plantago lanceolata* L. |  |
| *Plantago major* L. |  |
| *Veronica chamaedrys* L. |  |
| *Veronica scutellate* L. |  |
| Celastraceae |  |
| *Parnassia palustris* L. | NS/CI |
| Amblystegiaceae |  |
| *Drepanocladus aduncus* (Hedw.) Warnst. |  |
| *Pseudo-calliergon lycopodioides* (Brid.) Hedenäs |  |
| Apiaceae |  |
| *Berula erecta* (Huds.) Coville |  |
| *Oenanthe lachenalia* Gmel. | CI |
| Rubiaceae |  |
| *Galium palustre* L. |  |
| Araliaceae |  |
| *Hydrocotyle vulgaris* L. |  |
| Lythraceae |  |
| *Lythrum salicaria* L. |  |
| Ericaceae |  |
| *Pyrolaro tundifolia* L. | CI |
| Elaeagnaceae |  |
| *Hippophaer hamnoides* L. |  |
| Hypnaceae |  |
| *Calliergonella cuspidata* (Hedw.) Loeske |  |
| Hypericaceae |  |
| *Hypericum tetrapterum* Fr. |  |
| Lamiaceae |  |
| *Mentha aquatica* L. |  |
| Iridaceae |  |
| *Iris pseudacorus* L. |  |
